# Encountering Anger in the Emergency Department: Identification, Evaluations and Responses of Staff Members to Anger Displays

**DOI:** 10.1155/2012/603215

**Published:** 2012-08-02

**Authors:** Cheshin Arik, Rafaeli Anat, Eisenman Arie

**Affiliations:** ^1^Department of Social Psychology, University of Amsterdam, Weesperplein 4, 1018 XA Amsterdam, The Netherlands; ^2^William Davidson Faculty of Industrial Engineering and Management at Technion City, Technion-Israel Institute of Technology, Haifa 32000, Israel; ^3^Department of Emergency Medicine, The Western Galilee Hospital, P.O. Box 21, Naharia 22100, Israel

## Abstract

*Background*. Anger manifestations in emergency departments (EDs) occur daily, interrupting workflow and exposing staff to risk. *Objectives*. How staff assess and recognize patients' angry outbursts in EDs and elucidate responses to anger expressions, while considering effects of institution guidelines. *Methods*. Observations of staff patient interaction in EDs and personal interviews of staff (*n* = 38) were conducted. Two questionnaires were administered (*n* = 80 & *n* = 144). Assessment was based mainly on regression statistic tests. *Results*. Staff recognizes two types of anger displays. Magnitude of anger expressions were correlated with staff's fear level. Staff's responses ranged from ignoring incidents, giving in to patients' requests or immediately calling security. When staff felt fear and became angry they tended to call security. Staff was more likely to ignore anger when incident responsibility was assigned to patients. *Discussion*. Anger encounters are differentiated according to intensity level, which influences interpretations and response. Organizational policy has an effect on staff's response. *Conclusions*. Staff recognizes anger at varying levels and responds accordingly. The level of danger staff feels is a catalyst in giving in or calling security. Call security is influenced by fear, and anger. Permanent guidelines can help staff in responding to anger encounters.

## 1. Background

The atmosphere in ED is usually stressful, especially among patients and escorts who always consider their medical problem as urgent, requiring immediate attention. People tend to be impatient and restless. Violent outbursts by patients and their escorts are common and put the safety of both patients and caregivers at risk [1–7]. The prevalence and frequency of anger encounters at hospitals are so extreme they have been defined as a hazard of the health profession [5]. Moreover, the consequences of encountering anger communication from patients and family members, has been found to lead staff to feel alienation [5] and are considered to be one of main the causes to burnout and stress [6]. The staff needs not only to care for and treat patients, but also must manage and deal with these angry outburst. A meticulous review of the literature found that the general responses of nurses' to patient aggression are similar across countries and cultures [6]. Beyond preventing patients' aggression, it is necessary to develop better ways to for nurses to cope with this alarming problem [6]. What is known today is that aggression and violence are prevalent in hospitals. Moreover, the impact of these incidents on the medical staff has taken on an inward focus, looking at intrapersonal effects. A study using qualitative methods only found that the staff either connects or disconnects with the patients after an angry outburst. It was found that the higher the self-efficacy and experience of the staff the better they were able to connect with aggressive patients [8]. Yet, little is known about the process the staff undergoes during the actual encounter.

When it comes to organizational perspectives, it was found that training regarding aggressive incidents and improving self-control and communication skills can reduce some of these incidents [9]. Yet, the effects of staff awareness to official guidelines and procedures have not been studied sufficiently.

## 2. Objectives

This project was undertaken in order to understand the process that staff members undergo when encountering anger from those for whom they are trying to provide care. Investigating the interaction between the medical staff and patients is crucial for understanding this disturbing phenomena. One of the goals of this study is to elucidate the types of responses adopted in cases of anger encounters in the ED and determine what guides these responses. A second goal is to test what leads to these responses, focusing on staff's evaluation and feelings regarding the anger encounter. Finally, the project evaluates the effects and influences of official institutional guidelines and training on responses to anger encounters.

## 3. Methods

The study was conducted at three large public hospitals. These hospitals are located in central and northern Israel. The study included a number of stages. A preliminary study was conducted where medical staff, including doctors, nurses, and receptionists in the ED were observed while they interacted with patients and visitors. Over 20 hours of observations were conducted. A list of possible reasons for anger and responses were recorded during each observation. This list was processed and checked for redundancy. Observations continued until saturation was achieved and observations no longer revealed additional reasons for anger. Moreover, 38 nurses were interviewed (26 female, mean tenure = 7.89 years, tenure range: a few months –30 years). This data was analyzed looking at novel and new information obtained from each interview and observation, as well as checking for the frequency of each answer. The preliminary study was seized once saturation was met and no new information appeared.

In the second stage of the study open-ended questions and template questionnaires were used to better understand this phenomenon and allow for statistical testing. Participants were asked to “Describe an event of encountering anger in your own professional past and the way you coped with it” this was accompanied with a number of template questions (*n* = 80, 62 females, mean tenure = 12.44 years, tenure range: a few months–years, 81% nurses, 10% other support staff, 9% doctors).

The results from both stages were used to create the central questionnaire (*n* = 144, 100 females, Mean tenure = 12.35, tenure range: a few months–37 year, Mean age = 36.23, age range: 19–67 years, 78% nurses, 12% other support staff, 10% doctors), which described a scenario (one of eight possible). The scenarios differed in only two factors: age of the aggressor (young or old) and expression of anger. Respondents were asked to assess a hypothetical staff member's impression of the angry man and the situation. Assessment of the level of danger the staff member was under, the responsibility for this angry outburst, and the emotion the staff felt were measured via a self-report questionnaire. In addition, respondents answered questions regarding the existence of official guidelines and procedures, as well as training received for dealing with anger and aggression. The last part of the questionnaire asked participants to indicate the likely response of the hypothetical staff member to the event.

**Statistics**

Assessment was based mainly on regression and multiple regression statistic tests. Significance was calculated by ANOVA. Correlation tests were performed according to Pearson exact test.

## 4. Results

From the preliminary studies it was found that the most common reason for angry outbursts pertained to the length of wait in the ED, where the average waiting time is roughly three hours. The medical staff expects expressions of discontent and anger from the patients and their escorts after two hours wait. Types of anger expressions that were identified ranged from hostile stares, shouts, to pounding on the counter. It was found that these anger displays differed significantly based on two aspects: loud and silent. Loud anger was harder to ignore and included shouts and pounding on the counter, whereas silent anger was less noticeable and included stares at the staff and uneasiness.

Using the main questionnaire we assed these finding qualitatively.  Table 1 presents the intercorrelations between study variables. By measuring the perception of staff as to how angry the patient was (on a scale of 1–7), it was confirmed that indeed two types of anger are identified by the staff. The two measurements were significantly different from one another (mean loud anger = 5.68, mean silent anger = 4.80, *F*(1,142) = 27.87, *P* < 0.001). Yet, both of these expressions were rated as significantly higher than the midpoint of the scale pertaining to how angry the angry patient was (loud anger and midpoint *t*(71) = 24.15,  *P* < 0.001; silent anger and midpoint *t*(71) = 9.33,  *P* < 0.0001).

### 4.1. Impact of Anger on the Staff's Feelings and Judgment

The expressions and magnitudes of anger were directly correlated with the staff's fear level. The fear level increased in direct relation to the loudness of anger expression (*F*(1, 142) = 16.47, *P* < 0.001, beta = 0.32, *P* < 0.001). The higher the intensity of anger that was displayed toward the staff the higher the anger and frustration the staff felt (*F*(1,141) = 22.48, *P* < 0.001, beta = 0.37, *P* < 0.001). The age of the displayer had an effect only at the level of dangers assessed (*F*(1, 142) = 16.27, *P* < 0.001, beta = 0.32, *P* < 0.001), yet it had no significant effect on other interpretations (patient's fault *F*(1, 142) = 2.33, n.s.; staff's anger and frustration *F*(1, 142) = 0.03, n.s.). Assessing responsibility for anger events (i.e., quantifying patient or escorts responsibility versus the responsibility of the staff for creating conditions that led to anger) was found to have a direct relationship with the intensity of anger displayed (*F*(1, 142) = 5.85, *P* < 0.05, beta = 0.20, *P* < 0.05).

### 4.2. Response Characteristics

**Figure 1:**
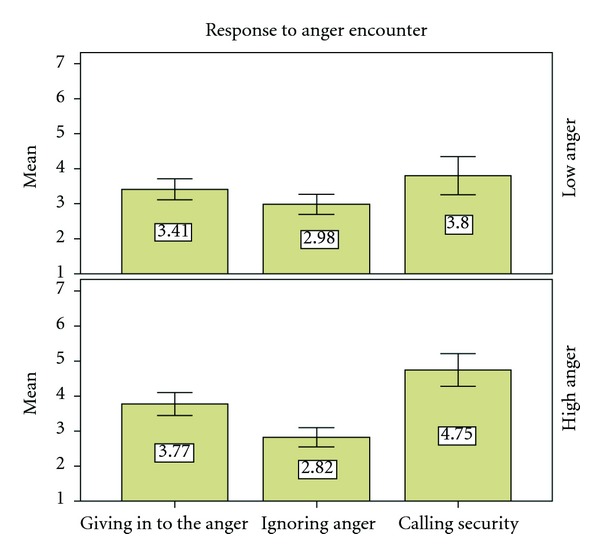
Response to anger encounter. The participants indicated on a 1–7 point scale the likelihood they would respond in one of the three ways (1) giving in to anger, (2) ignore the anger, (3) call security. Calling security is the only significant difference between the high and low anger (*F*(1, 141) = 6.91, *P* < 0.05).

It ranged from ignoring the incident, giving in to the request of the patient or escort or immediately calling security (see Figure 1).

#### 4.2.1. Ignoring the Anger Incident

The staff was more likely to ignore the anger when they assigned the responsibility for the incident to the patient (*F*(1,140) = 3.40, beta = 0.15,  *P* = 0.07). Moreover, when there were clear written protocols on how to behave in anger encounters or whenever the team underwent appropriate training to deal with anger and violent expressions, there was a significantly lower likelihood of the staff ignoring the anger incident (*F*(1,140) = 5.06, beta = −0.19,  *P* < 0.05).

#### 4.2.2. Giving in to the Anger

The higher the perception of threat, the more likely the staff was to give in to the anger (*F*(1,140) = 12.54, beta = 0.29, *P* < 0.01). Moreover, the organizational guidelines, protocols, and training had an effect on this response as well. The clearer the guidelines and protocols were, the more likely the staff was to respond by giving in to the request of the angry person (*F*(1, 140) = 5.78, *P* = 0.02). When testing their joint effect both of these variables were found to have a significant impact on giving in to the anger (*F*(2,139) = 8.09, beta threat = 0.26, *P* < .001, beta policy = 0.15, *P* = 0.07).

#### 4.2.3. Calling Security

The higher the threat that staff perceived, the higher the likelihood that the response would be to call security (*F*(1,140) = 60.02, beta = 0.55, *P* < 0.001); the more angry and frustrated the staff felt, the higher the likelihood that they would call security (*F*(1,140) = 35.33, beta = 0.45, *P* < 0.001). Awareness of organizational guidelines policy and protocol influenced the likelihood of calling security (*F*(1, 140) = 3.87, beta = 0.13, *P* = 0.05). When testing the joint effect of all three variables we found that only the level of threat and staff anger and frustration were still significant (*F*(3,138) = 22.88, beta threat = 0.41, *P* < 0.001, beta anger and frustration = 0.21, *P* = 0.02, beta policy = 0.11, n.s.).

### 4.3. Impact of Judgment among the Staff

Judgment and assessments of the situation constitute a bridge that mediates between the type and intensity of the anger event and the choice of how to cope with it. Assessment of danger, aggressor responsibility, and feelings of anger and frustration mediated the relationship between encountering anger displays and the various response types. Giving in to the anger (i.e., moving towards the aggressor) had an indirect effect on all meditating variables significant at the 90% level as indicated by the Confidence Interval (CI) (CI 0.03 to 0.39), where assessment of danger alone mediated the relationship at the 95% level (CI 0.06 to 0.48). Ignoring the anger was mediated only by the assignment of responsibly to the aggressor at the 90% level (CI 0.01 to 0.26). Calling security was mediated by all variables at the 95% level (CI.44 to 1.40) and by the variables of assessment of danger at the 95% level (CI 0.20 to 0.99). Calling security was also mediated by feelings of anger and frustration from the staff at the 95% level (CI 0.01 to 0.76).

It is important to note that role, tenure, and gender of the medical staff had no impact on the results. The sample was not balanced, yet when taking these different characteristics into account the results were not affected.

## 5. Discussion

Anger encounters between patients (and/or their escorts) and medical staff are vastly prevalent in emergency departments [1–5]. Every day medical staff members need to cope with anger directed towards them by the same people for whom they are expected to provide treatment and care. Most studies in this area have dealt with measuring the broadness of the phenomenon and the internal and personal impact it has on the staff. The current study shades light on the process the staff members undergo when encountering low-level aggression and anger display. Specifically, the identification and evaluation of anger were tested to see how it impacted responses to the perpetrator of aggression.

In this respect this paper has important implications that are both practical and theoretical. Understanding how the staff assesses anger encounters and responds to them, as well as understanding the organizational aspects concerning acceptable interventions when encountering anger or aggression is crucial if as we search for an effective manner in which to cope with the difficult reality of intense anger and aggression that ED staff must cope with on a daily basis. Our results indicate that when staff members encounter anger they evaluate two main questions: am I under danger? And who is to blame for the anger: is it the staff or the patient? These two evaluations influenced the staff's responses where danger led staff to either give in to the patients with their request or call security. Assignment of responsibility to the patient led staff to ignore the angry encounter while staff anger and frustration increased the chances that security would be called. Another variable that influenced response was the awareness the staff member had concerning the organizational policy for dealing with anger encounters. The higher the awareness, the lower were the chances of ignoring the anger and the higher the chances were for giving in to the request of the patient. Calling security was found to be more robustly linked to the assessment of danger level and the fear sensation of the staff than to the organizational policy (see Table 1).

An additional aspect that has been studied by others is a comparison of the angry or aggressive outbursts of normative people versus those who have been diagnosed as mentally ill (e.g., [9]). The current study has looked at normal functioning patients. We argue that the aggressive acts of mentally ill individuals are different from those of normal functioning members of society. Our results and analyses refer to patients who are assumed to be normally functioning members of society who are at the ED due to an emergency.

On a theoretical basis this paper contributes to the interpersonal and social impact of anger that has recently started to receive attention in organizational literature [10–12]. Hareli and Rafaeli, who have meticulously described the social aspects of emotion and the process involved in interactions dealing with emotion, called for more work to be done on specific cases of emotional display while following the entire emotional interactive process they entail. This project furthers this line of research and provides insights into the social aspect of anger encounters.

While the policy regarding zero tolerance to violence and aggression is on the rise, this project shows that the display of anger has an impact on staff members even in the low spectrum of aggression. Moreover, anger encounters can be differentiated in terms of intensity which influences both interpretations and response to these encounters. There might be room to consider when it is that anger or aggression has crossed the line of appropriateness and when it might still be legitimate and acceptable.

On a more practical note it is clear that the staff feels threatened when encountering anger of others. Although this is not a new finding and other studies have found this effect, the current study illustrates that feelings of threat impact the response the staff chooses when encountering anger. Ways of reducing this fear should be considered. This might involve training the staff and adding more security personnel. Moreover, anger and frustration also arise when encountering angry patients. Staff should be given a legitimate place to voice their frustration and vent their own anger. Most importantly, it was found that the organization could shape the responses the staff had by providing guidelines, protocols, and training to the staff.

One of the strengths of this study is that it used mixed methods to study and investigate this phenomenon. Most studies to date use one of the two methods [3, 8]. Using both qualitative and quantitative methods offers higher ecological validity and allows to reap the benefits of the richness of qualitative data and the systematic testability of quantitative data. The current study used the qualitative study to develop a quantitative tool that was falsifiable.

We should note that this study like all others has limitations as well. The study uses self-report data and not actual behavior. Obtaining actual behavior in these situations is almost impossible. By coding and looking at real incidents and behavior, self-report biases would be eliminated. We have tried to create the most realistic scenarios and accompanying questions by observing and interviewing the staff. One possible way to do so in future would be by analyzing data from surveillance cameras that are currently present in many ED. Another limitation to our study is related to our sample which was not balanced in terms of the various roles at the ED and mostly focused on nurses. Past studies have found variation in exposure and responses to aggression based on position and seniority [12]. Future studies should take this factor into account. The prevalence of aggression and violence of mentally ill patients is high. In this study we have decided not to focus on this population. It might be that the staff has different evaluations when it comes to encounters of anger from these patients, this questions still remains untested.

## 6. Conclusions

1. Permanent guidelines should be built in to hospital policies to deal with anger incidents in the ED. Our results elucidate how these guidelines still have an effect even when controlling for other factors such as intensity of anger encounter and staff interpretations.
2. The staff recognizes anger at varying levels and responds accordingly.
3. The level of danger staff feels after encountering anger is a catalyst in the staff's response of giving in or calling security when they encounter anger.
4. Calling security is influenced by fear, frustration, and anger. Limiting this response would depend on attending to the staff needs for security and a legitimate way to voice anger and frustration.

## Figures and Tables

**Table 1:** Correlations between all the study variables. For example row 1 shows all the correlations with “Patient anger intensity” so the correlation between this variable and ignoring is 0.01.

	*M*	SD	1	2	3	4	5	6	7	8
(1) Patient anger	5.24	1.08	(0.79)							
(2) Interpretation of threat	3.37	1.33	0.45^∗∗^	(0.92)						
(3) Interpretation of patient's responsibility	2.49	0.94	0.32^∗∗^	0.44^∗∗^	(0.76)					
(4) Staff's anger and frustration	3.74	1.31	0.46^∗∗^	0.58^∗∗^	0.36^∗∗^	(.70)				
(5) Organizational policy and guidelines	3.32	1.47	0.07	0.17^∗^	0.05	0.03	(.81)			
(6) Giving in to the anger (Move Toward)	3.59	1.34	0.22^∗∗^	0.29^∗∗^	0.08	0.16^†^	0.20^∗^	(0.53)		
(7) Ignore	2.90	1.18	0.01	0.01	0.15^†^	0.11	−0.19^∗^	−0.12	(0.65)	
(8) Calling security	4.27	2.18	0.29^∗∗^	0.55^∗∗^	0.26^∗∗^	0.45^∗∗^	0.16^†^	0.11	0.02	—

Means (*M*), standard deviations (SD), and intercorrelations among study variables.

*n* = 144. Reliabilities are on the diagonal; ^†^ *P* < 0.01; *P < 0.05; **P < 0.01.

## Abbreviation

n.s: Nonsignificance.
